# Abnormal lymphatic system function in patients with chronic neck and shoulder pain: neuroimaging evidence from DTI-ALPS

**DOI:** 10.3389/fnmol.2026.1707858

**Published:** 2026-04-02

**Authors:** Jiayu Yang, Tianci Liu, Ruhui Xiao, Yu Fu, Xiaowei Kong, Zhiqiang Qiu, Xiaoxue Xu

**Affiliations:** 1 College of Medical Imaging, North Sichuan Medical University, Nanchong, China; 2 Department of Radiology, Affiliated Hospital of North Sichuan Medical College, Nanchong, China

**Keywords:** chronic neck and shoulder pain, CNSP, DTI-ALPS, lymphatic system, neuroimaging

## Abstract

**Objective:**

Chronic neck and shoulder pain (CNSP) is frequently associated with structural and functional brain damage that progressively worsens over time. The glymphatic system (GS), a gel-like extracellular fluid in the brain, plays a crucial role in clearing waste products and maintaining interstitial fluid homeostasis. However, its relationship with CNSP remains unclear. This study employed diffusion tensor imaging along perivascular spaces (DTI-ALPS) to investigate functional changes in the lymphatic system among patients with chronic neck and shoulder pain, while exploring potential correlations with clinical outcomes.

**Methods:**

This study enrolled 20 patients with CNSP and 20 healthy controls (HCs). All participants underwent standardized scans using a 3.0T MRI scanner. By calculating the bilateral DTI-ALPS indices of the two groups, we compared the functional status of GS between the groups and further established its correlation with clinical indicators.

**Results:**

Following a permutation test (10,000), the DTI-ALPS index in the left cerebral hemisphere of the CNSP group was significantly lower than that in the HCs group (*P* = 0.0413). Within the CNSP group itself, the left hemisphere showed a more pronounced reduction compared to the right side (*P* = 0.0012). Partial correlation analysis revealed that after excluding disease duration as a variable, VAS scores demonstrated a significant correlation with left hemisphere DTI-ALPS index (*r* = −0.651, *P* = 0.003). When VAS was excluded from the analysis, disease duration showed a statistically significant association with left hemisphere DTI-ALPS index (*r* = −0.727, *P* < 0.001).

**Conclusion:**

Our findings indicate that CNSP may lead to abnormal GS function in the left hemisphere as the disease duration prolongs and pain intensity increases.

## 1 Introduction

Neck and shoulder pain is a common chronic condition associated with the imbalance of anterior and posterior spinal muscle strength due to prolonged traumas, resulting in various degrees of discomfort, swelling, and restricted motor function among patients (Raja et al., 2020). When symptoms persist for more than three months, it is termed chronic neck and shoulder pain (CNSP) (Stanton et al., 2016). Incomplete statistics indicate that the annual incidence of chronic neck and shoulder discomfort varies between 10.4% and 21.3%, with a greater frequency observed in women, the elderly, and individuals engaged in office and computer-related occupations (Frobin et al., 2001). As the disease develops, patients experience a deterioration in neurological function, impacting their attention, mood, cognitive status, bodily activity, and overall quality of life (Denkinger et al., 2014). Presently, conservative treatment serves as the primary clinical approach for CNSP, encompassing oral medicine, acupuncture, massage, and exercise therapy; nevertheless, its efficacy is suboptimal (Qiu et al., 2025). Surgery is beneficial for CNSP mostly caused by cervical radiculopathy; nevertheless, it is not the recommended treatment due to associated risks. Consequently, prompt diagnosis and timely standardized therapies are essential for alleviating pain and managing the condition.

The etiology of CNSP is complex, with MRI commonly revealing structural and functional alterations in brain regions associated with pain. The constant discomfort in CNSP patients leads to widespread abnormalities in neuronal activity across several brain regions, resulting in disruptions in neuroplasticity, electrophysiology, and neurochemical functional connections (Otti et al., 2013). These regions primarily engage in the integration and processing of nociceptive signals, mediating pain sensitivity, cognitive memory, affective processing, emotional control, and speech motor impairments in patients (Qiu et al., 2025; Yu et al., 2017). Furthermore, long-term CNSP patients demonstrate elevated pain awareness relative to healthy individuals (Zhang et al., 2024), and ongoing pain signals to the cerebral cortex may result in cumulative harm to brain microstructures (Zhao et al., 2013), accompanied by pain sensitization. This process subsequently influences the architecture of adjacent brain regions, ultimately resulting in deficits in cognitive processing, memory, and emotional regulation.

It is well known that the brain lacks the traditional lymphatic network found in other peripheral tissues. Consequently, GS emerges as the pivotal mechanism for waste clearance and regulation of interstitial fluid homeostasis within the brain parenchyma (Rasmussen et al., 2018; Iliff et al., 2012). GS in the brain is a sophisticated network of perivascular spaces (PVS), primarily composed of astrocytes with perinuclear extensions that exhibit high expression of Aquaporin-4 water channels (AQP4), facilitating the convective exchange of cerebrospinal fluid (CSF) and interstitial cerebral fluid (ISF) constituents (Vittorini et al., 2024). The CSF-ISF complex serves as the primary vehicle for waste clearance within the brain. Cerebrospinal fluid produced by the choroid plexus within the ventricles initially accumulates in the ventricles, PVS, and subarachnoid spaces (Vittorini et al., 2024). It then enters the brain tissue via the para-arterial space, where it mixes with ISF. Subsequently, it passes through AQP4 channels on the terminal processes of astrocytes into the perivascular spaces (Iliff et al., 2012), ultimately draining into the cervical lymphatic vessels (Tarasoff-Conway et al., 2015).

Previously, the predominant methods for evaluating GS function involved intrathecal administration of a contrast agent, with the clearance time of the tracer from the brain determined by monitoring the temporal variations in image signal intensity, thereby indirectly indicating the functionality of the cerebral lymphatic system (Wang et al., 2023). However, this approach was met with patient resistance due to its invasive nature, significant radiation exposure, and the necessity for multiple drug administrations. Diffusion tensor image analysis along the perivascular space (DTI-ALPS) is a technique introduced by Taoka et al., 2017 for evaluating GS function, characterized by its ease of use, non-invasiveness, and high reproducibility (Taoka et al., 2017). The ALPS index is calculated without contrast enhancement, utilizing standard MRI sequences, including magnetic susceptibility-weighted imaging (SWI) and diffusion tensor imaging (DTI) to derive the relevant phase maps, color-coded fractional anisotropy (FA) maps, and diffusion maps along the *x*-, *y*-, and *z*-axes (Liu et al., 2024). The *x*-axes represents the medullary vein, which traverses perpendicularly to the ventricular wall at the level of the lateral ventricle body; the *x*-axes also denotes the pial vein. The fibers traverse perpendicularly to the ventricular wall; the upper longitudinal bundle, representing the association fibers, is designated as the *y*-axes, primarily oriented in the anterior-posterior direction. Beyond the association fibers, subcortical fibers extend in the left-right direction within the subcortical region, while projection fibers adjacent to the lateral ventricles predominantly travel in the cephalocaudal direction, defined as the *z*-axes. When a corresponding alteration is detected in both fiber bundles, it is deemed to be wholly or at least partially attributable to pathological changes in the PVS (Vittorini et al., 2024). DTI-ALPS index = mean (Dxproj, Dxasso)/mean (Dyproj, Dzasso) (Taoka et al., 2017). The lower the ALPS index, the worse the function of the GS.

Previous studies have found that various painful conditions may be associated with varying degrees of lymphatic circulation abnormalities, though significant discrepancies persist between different research conclusions. In migraine, Zhang et al. (2023) discovered that chronic migraine patients (CM) exhibited higher DTI-ALPS indices than both healthy controls (HCs) and episodic migraine patients. However, no significant difference in DTI-ALPS indices was observed between the episodic migraine group and HCs. Similarly, in line with Zhang’s findings, studies by Lee et al. (2022) (patients with migraine with and without aura), Ren et al. (2025) (episodic migraine), Cao et al. (2024) (episodic migraine) and others did not observe significant differences in DTI-ALPS indices between migraine patients and healthy controls. However, Cao et al.’s (2024) subsequent investigations revealed markedly elevated right-sided diffusion kurtosis imaging analysis along the perivascular space (DKI-ALPS) indices in the patient cohort. Concurrently, Ren et al.’s (2025) application of dynamic contrast-enhanced MRI (DCE-MRI) further confirmed increased PVS burden within the superior semi-ellipsoid region of episodic migraine patients during DCE-MRI, providing supplementary evidence for lymphatic system involvement in migraine pathology. In cancer pain research, Wang et al. (2022) found that the diffusion rate and ALPS index in the cancer pain (CP) group were significantly lower than those in the painless cancer (PLC) group and HCs group, and significantly increased after analgesic intervention, further confirming the close association between lymphatic dysfunction and pain. In classic trigeminal neuralgia, both studies found a significant decrease in bilateral DTI-ALPS indices, accompanied by neurofluid dysregulation (Chen et al., 2025) and hippocampal volume changes (Hou et al., 2025). Moreover, reduced brain DTI-ALPS indices were also observed in knee pain and fibromyalgia (Valdes-Hernandez et al., 2025; Tu et al., 2023).

So far, the precise role of the GS in chronic pain remains controversial, and no studies have yet been published regarding the significance of altered GS function and the DTI-ALPS index in CNSP. This study aims to evaluate the GS function in CNSP patients using the DTI-ALPS index, explore novel neuroimaging markers indicative of pain, and provide fresh insights to deepen understanding of chronic pain disorders and guide therapeutic practice.

## 2 Materials and methods

### 2.1 Participants

From May 2023 to June 2024, 44 people were recruited from the Affiliated Hospital of North Sichuan Medical College, including 24 patients with CNSP and 20 healthy individuals matched by sex, age, and dominant hand, who did not have any neurological or pain issues. The diagnosis of CNSP disease was conducted by two seasoned pain physicians at the Affiliated Hospital of North Sichuan Medical College, adhering to the criteria set forth in the International Classification of Diseases, 11th edition (ICD-11) (Treede et al., 2015) for chronic pain categorization.

CNSP group: Inclusion criteria included: (1) persistent neck and shoulder pain symptoms exceeding three months; (2) individuals aged 18 years or older with right-hand dominance; (3) absence of neurological or psychiatric disorders or cognitive impairments; (4) no pain in other anatomical regions; and (5) no pain-related interventions within two weeks preceding the examination. The exclusion criteria included (1) significant cranial and cerebral trauma; (2) severe systemic illnesses, including tumors and tuberculosis; (3) prior neck surgery or congenital cervical spine anomalies; (4) pregnancy or lactation; and (5) the presence of metal implants or an inability to endure extended MRI procedures.

HCs group: Inclusion criteria included: (1) previous physical fitness, no intracranial lesions or neurodegenerative or other diseases affecting cognition; (2) no history of acute neck trauma or cervical spine tumors; and (3) no contraindications to MRI. Exclusion criteria included: (1) a history of chronic pain; and (2) the presence of psychiatric disorders such as depression, mania, and schizophrenia.

Prior to the commencement of this investigation, each participant was apprised of the experimental protocol and associated details, and all participants furnished signed informed consent. The research received clearance from the Ethics Committee of the Affiliated Hospital of North Sichuan Medical College, designated by approval number 2023ER95-1, and was executed in compliance with the Declaration of Helsinki.

### 2.2 Assessment of clinical indicators

Prior to MRI scanning, data on gender, age, and pain duration of the participants were gathered (not recorded in the healthy group), and the CNSP patients were evaluated for pain on a visual analog scale (VAS), with patients rating their pain experiences on a range from 0 to 10. 0: complete absence of pain sensation. 1∼3: signifies minor pain, typically manageable by the patient and not impacting daily activities. 4∼6: signifies moderate pain, causing considerable discomfort that may impact sleep and everyday activities. 7∼9: signifies intense pain that is excruciating and intolerable, significantly disrupting sleep and everyday activities. 10: denotes extreme pain, typically unbearable for individuals and necessitating immediate intervention.

### 2.3 Image acquisition

A 3.0T MRI scanner (MAGNETOM Skyra, Siemens, with a standard 20-channel head coil) was utilized to scan all participants in this investigation. Sequence usage: Three-dimensional (3D) Magnetization Prepared Rapid Gradient Echo (MP-RAGE) was utilized to acquire T1 high-resolution structural images with a repetition time (TR) = 2,240 ms; inversion time (TI) = 1,130 ms; data matrix = 256 × 256; field of view (FOV) = 256 mm × 256 mm; slices = 192; slice thickness = 1 mm without gap. Diffusion magnetic resonance imaging (dMRI) data, with a 2 mm isotropic resolution, were acquired using an echo planar imaging (EPI) sequence featuring a multiband factor (MB) = 4; TR = 10,500 ms; echo time (TE) = 92 ms; data matrix = 128 × 128; FOV = 256 mm × 256 mm; slices = 72; 30 non-coplanar diffusion directions with b = 1,000 s/mm^2^; 5 AP (Anterior-to-Posterior) and 5 PA (Posterior-to-Anterior) images with b = 0 s/mm^2^; slices = 72; slice thickness = 2 mm without gap.

Before the examination, individuals with early pregnancy and claustrophobia were excluded; participants and their relatives were cautioned against bringing magnetic metals into the MRI room; participants were informed that the examination would last 20 min and that they should maintain relaxation, stillness, and steady breathing throughout the procedure. Throughout the assessment, all participants were positioned supinely, secured with foam around the head, and equipped with earplugs to mitigate noise-related pain. If the subject cannot withstand the MRI examination, he/she should raise a hand to signal this inability.

### 2.4 Data preprocessing

(1) Data quality control: Resolution, number of gradient directions, b-value, signal-to-noise ratio, artifacts, and head motion were checked in the imaging data.
(2) Format conversion: The collected MRI data were converted from DICOM to NIFTI format using the dcm2niix tool.
(3) Noise correction: Thermal noise correction was performed in MRtrix3 software using Marchenko-Pastur principal component analysis.
(4) Gibbs ringing correction: Fixing errors caused by cutting off k-space or limited image sampling in MRtrix3 software (Kellner et al., 2016).
(5) Geometric distortion correction: This process involves correcting EPI geometric distortion by encoding b = 0 data in opposite phase directions, AP and PA, using the topup tool in the FSL software (Andersson et al., 2003).
(6) Image correction: The eddy tool in the FSL software was used to correct (Andersson et al., 2016) the aberrations and distortions caused by eddy currents and head motion; the intensity inhomogeneities of the diffusion MRI images were corrected in the ANTs software by using the N4BiasFieldCorrection tool.
(7) Removal of redundant structures: Non-brain structures such as scalp and skull were removed using the Brain Extraction Tool (BET) in the FSL software to preserve the intact cranial and brain tissues to minimize experimental errors.
(8) Tensor estimation: The tensor model of each voxel was derived using the DTIFIT tool in the FSL software to calculate the FA and mean diffusivity (MD).

### 2.5 Calculation of the DTI-ALPS index

The DTI-ALPS approach primarily utilizes the diffusivity of water molecules, as determined by the diffusion tensor technique, to evaluate the flow of water molecules into the PVS. In Taoka et al. (2017) originally proposed that DTI-ALPS may evaluate the functionality of the GS, indicating that a lower ALPS index correlates with diminished lymphoid system function.

Alignment to standard space: FA maps and diffusivity maps for *x*, *y*, and *z*-axes were generated in FSL software using the dtifit tool, and the generated FA images were aligned using the JHU-ICBM-FA template, and then the flirt tool was utilized to apply the transformation matrix to all the diffusivity maps.

ROI delineation: First, at the level of the lateral ventricular body, the projection fibers and associated fibers were identified as the superior corona radiata (SCR) and superior longitudinal fasciculus (SLF) (based on the images on which the JHU-ICBM-FA template was aligned); second, based on the JHU-ICBM-DTI-81-White Matter Labeling Atlas, the bilaterally outlined SCR and SLF regions on the diffusivity maps (5 mm in diameter); finally, the resulting ROIs coordinates were: left SCR (116, 110, 99), left SLF (128, 110, 99), right SCR (64, 110, 99) and right SLF (51, 110, 99) (Liu et al., 2024), and the Dxx, Dyy and Dzz diffusion coefficient values of the SLF and SCR in the region of the bilaterally outlined ROIs were to be automatically output in the FSL software.

**FIGURE 1:**
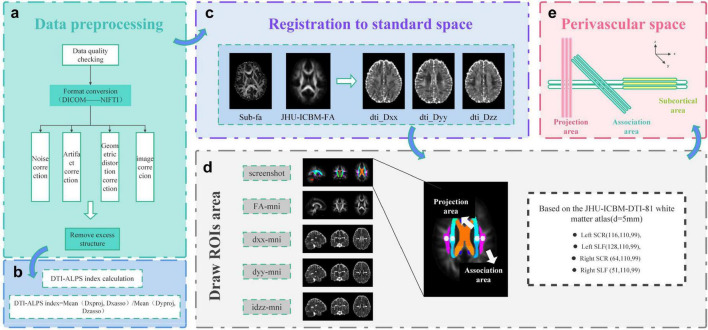
Schematic diagram of calculation process of DTI-ALPS method for perivascular space diffusion tensor imaging. **(a)** Data preparation process: Conducts quality inspection on acquired images, corrects noise, artifacts, geometric distortions, eddy currents, and head movement-induced distortions, removes excess tissue to facilitate subsequent analysis. **(b)** Perform DTI-ALPS index calculation using the formula: DTI-ALPS index = Mean (Dxproj, Dxasso)/Mean (Dyproj, Dzasso). A lower DTI-ALPS index indicates poorer function of the cerebral lymphatic system. **(c)** Aligns generated FA maps with diffusion tensor images in x, y, and z directions using the JHU-ICBM-FA template. **(d)** Defines ROIs coordinates (d = 5 mm) based on the JHU-ICBM-DTI-81 white matter atlas for ROC region delineation: Left SCR (116, 110, 99), Left SLF (128, 110, 99), Right SCR (64, 110, 99), and Right SLF (51, 110, 99). **(e)** Illustrates the relationship between perivascular spaces and fiber orientation: the *x*-axes represents subcortical fibers (yellow), the *y*-axes represents association fibers (green), and the *z*-axes represents projection fibers (pink), with all axes being mutually perpendicular.

(3) Calculation of DTI-ALPS index: The Dxx, Dyy, and Dzz diffusivity values of bilateral SLF and SCR are used in the formula DTI-ALPS index = mean (Dxproj, Dxasso)/mean (Dyproj, Dzasso) to derive the ALPS value (Figure 1).

### 2.6 Statistical analysis

(1) Data homogeneity test: Pearson’s chi-square test was used to compare the gender differences between the groups, and an independent samples *t*-test was used to compare the differences in age, pain duration, and VAS scores.
(2) Comparison of DTI-ALPS values: The permutation test (10,000) was used to compare the DTI-ALPS values of the ipsilateral cerebral hemispheres between CNSP group and healthy group and the DTI-ALPS values of both cerebral hemispheres within the group, and at the same time, age and gender were introduced into the model as covariates to control for their effects on the statistical results (*P* < 0.05).
(3) Correlation analysis: In order to exclude the influence of the third variable, partial correlation analysis was used to investigate the relationship between DTI-ALPS, pain duration, and VAS scores in patients with CNSP. The quantitative relationship between them was analyzed by linear regression analysis, which was statistically significant at *P* < 0.05.

## 3 Results

### 3.1 Demographics and clinical data analysis

Twenty CNSP patients and 20 healthy individuals were ultimately included in this study. In the CNSP cohort, two patients were eliminated due to suboptimal image quality resulting from excessive head movement during scanning, and two others terminated the scanning prematurely due to intense discomfort. Table 1 indicates that patients in the CNSP group exhibited an illness duration of 38.1 ± 10.331 months and a VAS score of 6.10 ± 1.021, with no significant differences in age and gender between the two participant groups (*P* > 0.05) (Table 1).

**TABLE 1:** Demographic data.

	CNSP (*n* = 20)	HCs (*n* = 20)	*P*
Gender			0.749
Male	9 (45.0%)	8 (40.0%)	
Female	11 (55.0%)	12 (60.0%)	
Age	53.6 ± 7.776	49.35 ± 11.744	0.185
Duration of pain	38.1 ± 10.331	–	
VAS	6.10 ± 1.021	–	

CNSP, chronic neck and shoulder pain; HCs, healthy controls; VAS, visual analog scale.

### 3.2 Rate of water diffusion in different directions between CNSP group and healthy control group

**FIGURE 2:**
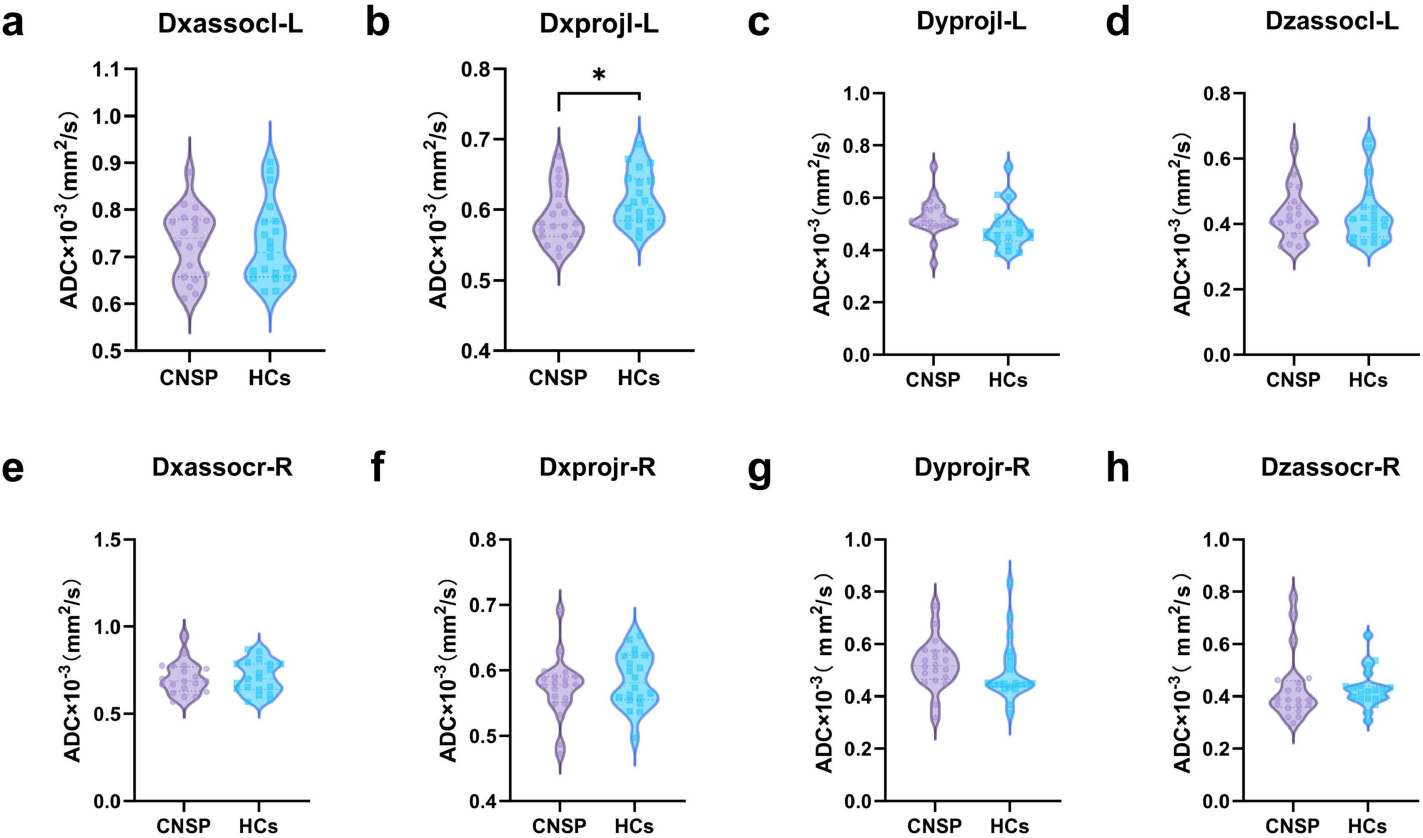
Comparison of water diffusion rates in different directions (x, y, z) between the bilateral cerebral hemispheres of CNSP and HCs populations. **(a–d)** represents water molecule diffusion rates in different directions of the left cerebral hemisphere, In the projection fiber regions of the left cerebral hemisphere, there was a statistically significant difference in the x-direction diffusion rate between CNSP and HCs, while **(e–h)** indicates those in the right cerebral hemisphere. Association area: Association fiber regions; projection area: projection fiber regions. HCs, healthy population; *P < 0.05.

Figure 2 shows the comparative diffusion rates of CNSP and HCs across all directions. In the Dxprojl-L direction, the diffusion rates for the two participant groups were 0.589 ± 0.040 and 0.615 ± 0.038, respectively. The diffusion rate of the CNSP group was significantly lower than that of the HCs group (*P* = 0.0436), while no statistically significant differences were noted in the other directions.

### 3.3 Correlation between diffusion rate of water molecules in all directions of ipsilateral cerebral hemisphere and VAS score

**FIGURE 3:**
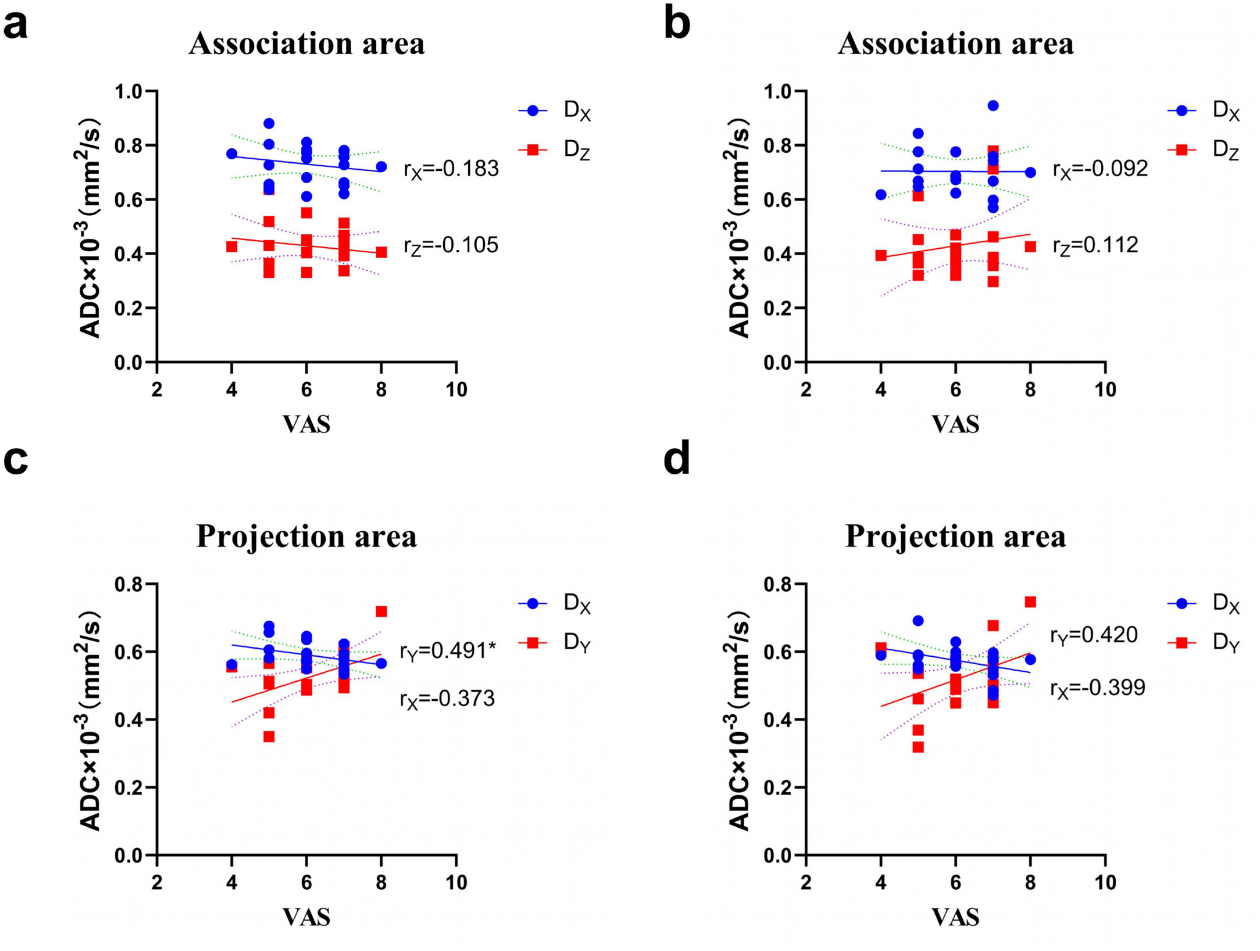
Correlation analysis between water diffusion rate in different directions of the ipsilateral cerebral hemisphere and VAS scores. **(a)** left hemisphere (Association area), **(b)** right hemisphere (Association area); **(c)** left hemisphere (Projection area), **(d)** right hemisphere (Projection area). The results show that in the left hemisphere’s projecting region along the *y*-axes direction, water diffusion rate correlates with VAS scores (*r* = 0.491, *P* < 0.05).*P < 0.05.

Figure 3 shows the relationship between the diffusion rate of water molecules in various directions inside the ipsilateral cerebral hemisphere and VAS values in CNSP patients. A notable positive correlation existed between the diffusion rate in the y-direction and the VAS score in the projection area of the left cerebral hemisphere (*r* = 0.491, *P* = 0.0278). However, no significant correlations were observed between the diffusion rate in the x-direction in the projection area of the left cerebral hemisphere, the x- and y-directions in the projection area of the right cerebral hemisphere, and the diffusion rates in the x- and y-directions in the association area of both cerebral hemispheres. No substantial connection was seen between diffusion rates and VAS scores in the x- and y-directions of the Association area region (*P* > 0.05).

### 3.4 Relationship between DTI-ALPS, pain duration, and VAS scores in CNSP patients

**FIGURE 4:**
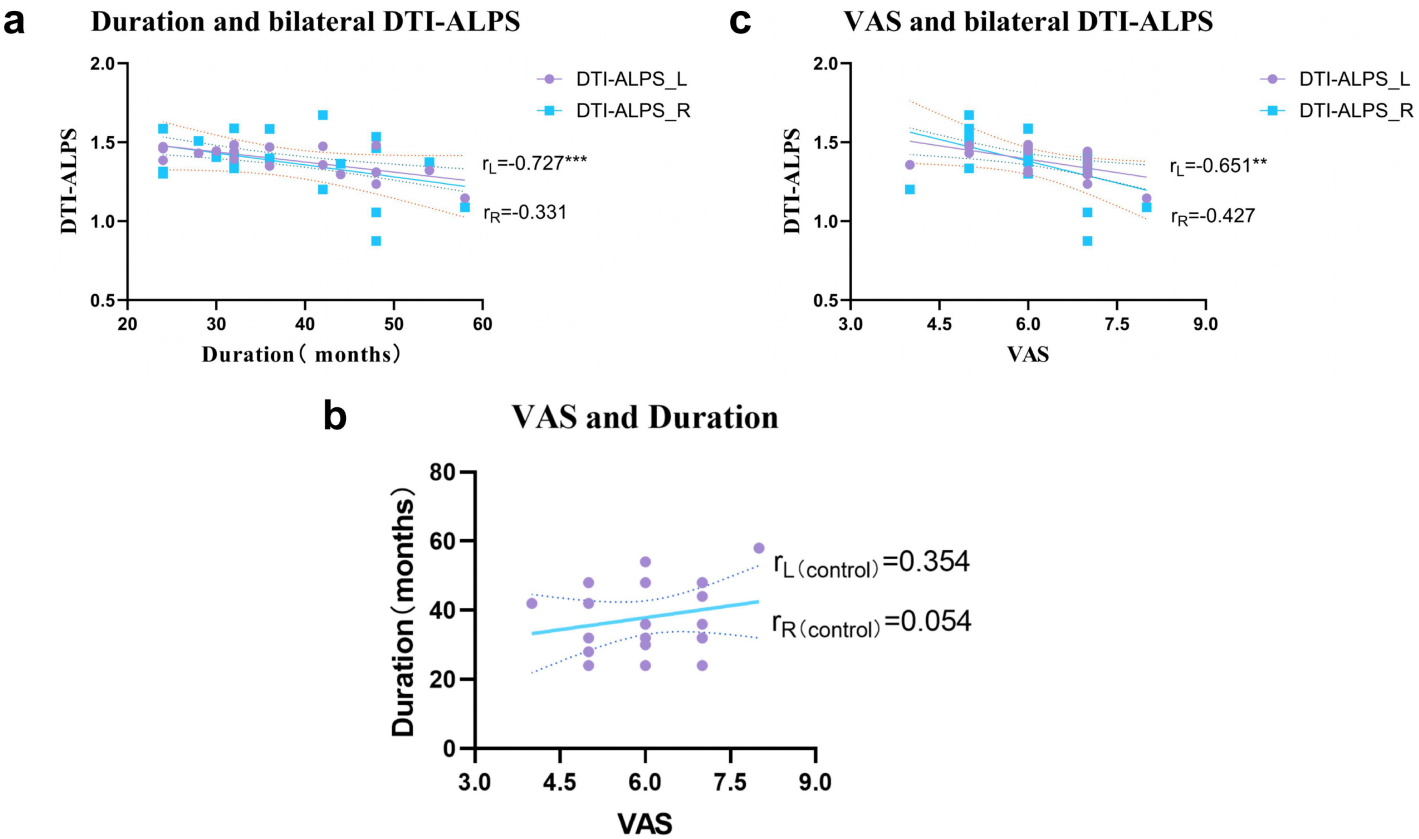
The relationship between DTI-ALPS, pain duration and VAS score in CNSP patients. **(a)** After excluding the variable of disease duration (months), a significant negative correlation between the DTI-ALPS index of the left cerebral hemisphere and VAS scores was observed (*r* = –0.651, *P* < 0.01); **(b)** after Excluded variables VAS, it was found that as the disease course extended (months), the DTI-ALPS index of the left cerebral hemisphere showed a significant decrease (*r* = –0.727, *P* < 0.001); **(c)** Excluded variables bilateral DTI-ALPS: excluded right side (*r* = 0.054), excluded left side (*r* = 0.354). *P < 0.05, **P < 0.01, ***P < 0.001; L, left cerebral hemisphere; R, right cerebral hemisphere.

The relationship between DTI-ALPS, pain duration, and VAS scores was examined by partial correlation, as illustrated in Figure 4. Upon excluding the influence of disease duration, VAS scores exhibited a significant correlation with the left cerebral hemisphere DTI-ALPS index (*r* = −0.651, *P* = 0.003). In contrast, when the impact of pain degree (VAS scores) was controlled, disease duration demonstrated a significant correlation with the left cerebral hemisphere DTI-ALPS index (*r* = −0.727, *P* < 0.001). However, VAS scores did not show a significant relationship with disease duration (*P* > 0.05).

### 3.5 Results of DTI-ALPS index comparison between CNSP group and healthy group

**FIGURE 5:**
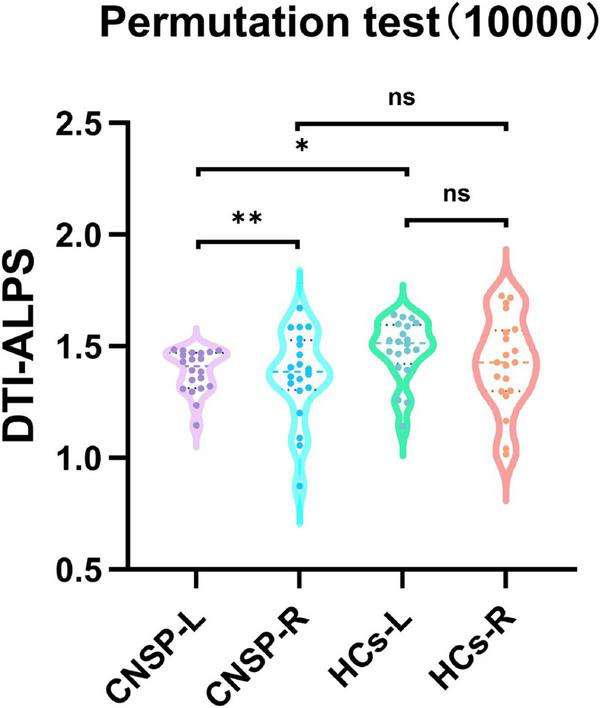
Comparison of DTI-ALPS values in the ipsilateral cerebral hemispheres and bilateral DTI-ALPS values within the group between CNSP group and healthy group. CNSP-L, left cerebral hemisphere of CNSP group; CNSP-R, right cerebral hemisphere of CNSP group; HCs-L, left cerebral hemisphere of healthy control group; HCs-R, right cerebral hemisphere of healthy control group; The results showed that compared with CNSP-R and HCs-L, the DTI-ALPS index of CNSP-L was significantly reduced. HCs, healthy people, *P < 0.05, **P < 0.01.

Figure 5 shows the results of comparing DTI-ALPS indices between the CNSP group and the healthy group after running 10,000 tests. A significant difference in DTI-ALPS indices was observed in the bilateral cerebral hemispheres of CNSP patients (*P* = 0.0012), while no statistically significant difference was detected in the healthy control group (*P* > 0.05). In the left cerebral hemisphere, CNSP patients exhibited a significant difference in the DTI-ALPS index compared to healthy controls (*P* = 0.0413), whereas this difference was not obvious in the right hemisphere (*P* > 0.05).

## 4 Discussion

In this study, we initially explored the role of GS function in CNSP by calculating the DTI-ALPS index in bilateral cerebral hemispheres and investigated its relationship with clinical features. It was found that GS function was severely impaired in the left cerebral hemisphere of CNSP patients and was significantly associated with clinical features. However, no abnormalities were observed on the right side.

Brain tissue is the most metabolically active tissue in the body. Previously, it was thought that the elimination of intracerebral waste primarily depended on CSF circulation; however, this method proved inadequate to satisfy metabolic demands until 2012, when Iliff et al. (2012) proposed the lymphatic system, thereby revealing the initial insights into cerebral metabolism. The elimination of waste products from the brain parenchyma occurs through their transport to the brain surface or the lateral ventricular wall. The lateral ventricular body, where the medullary vein is perpendicular to the body wall and aligned with the *x*-axes, has been recognized as a potential conduit for the movement of substances within the brain, coinciding with the orientation of the PVS. The diffusion capacity of water molecules at this site is influenced by various white matter fibers, and the geometric removal of vectors can be accomplished through the orthogonalization of projection fibers (*z*-axes) and association fibers (*y*-axes) along the x-direction, thereby assessing the water molecule diffusion capacity solely in the x-direction (Taoka et al., 2024). The DTI-ALPS index is primarily employed to evaluate the macroscopic movement of water molecules in the PVS direction, thereby reflecting GS activity laterally. It demonstrates strong stability and observational consistency, and its non-invasive nature has facilitated its extensive application in clinical practice (Liu et al., 2024).

GS serves as a pivotal mechanism for waste removal and regulation of interstitial fluid homeostasis within the brain parenchyma. The clearance of metabolic waste products in the brain predominantly occurs during sleep, particularly during non-rapid eye movement (NREM) sleep, whereas chronic pain frequently coexists with sleep disturbances (Yan et al., 2021). Valdes-Hernandez et al.’s (2025) research indicates that chronic widespread knee pain accompanied by diminished sleep quality significantly impacts DTI-ALPS signals in the left cerebral hemisphere, thereby affecting the brain’s ability to clear metabolic waste. Aligns with our findings, which indicated that left hemisphere DTI-ALPS was diminished in patients with CNSP and exhibited a significant positive connection with pain intensity and disease duration, whereas the difference in the right hemisphere was not statistically significant. A separate investigation into fibromyalgia revealed that chronic pain frequently correlates with an augmented choroid plexus volume (CPV) (Tu et al., 2023), which leads to diminished CSF production and permeability (Redzic et al., 2005). However, additional studies have not identified a relationship between sleep disorders and CPV enlargement (Kikuta et al., 2024), indicating that GS disturbances in chronic pain may stem from factors beyond sleep disorders. Comprehensive research indicates that noradrenaline plays a pivotal role in the transition from acute to chronic pain (Goldman et al., 2020), with pain exacerbating GS injury through activation of the adrenergic system and adrenal response, particularly noradrenaline. This activation may reduce venous return by influencing the expansion of the interstitial space (Valdes-Hernandez et al., 2025), vasoconstriction, and microglial activation, thereby inducing alterations in cerebral cellular activity and metabolism, which in turn promote cardiovascular disease and endothelial dysfunction (Tian et al., 2024; Malcangio, 2019). In healthy individuals, noradrenaline primarily suppresses lymphatic system function during wakefulness, with its secretion decreasing during sleep; however, in patients with chronic pain, adrenaline levels remain persistently elevated across all states, including both the resting state and pain states (Barbosa et al., 2024). Moreover, microstructural alterations in the brains of chronic pain sufferers may exacerbate GS disease damage, manifesting as changes in gray matter volume and abnormalities in white matter functional connectivity (Coppieters et al., 2016). This arises from neurofluid dysregulation, which impedes the excretory function of the endothelial cell system within deep brain tissues, thereby further diminishing the disease’s capacity to clear metabolic waste (Chen et al., 2025).

Interestingly, in previous migraine studies, Zhang et al. (2023) noted that during the chronic progression of migraine, the DTI-ALPS index increases, with a marked elevation on the right side. This observation may be closely associated with altered vascular reactivity linked to the pathophysiology of migraine. Studies have documented patterns of cerebral hyperperfusion in migraine sufferers (Bai et al., 2022). Elevated levels of CGRP, glutamate, and pro-inflammatory cytokines have been detected in the cerebrospinal fluid of patients (Kaag Rasmussen et al., 2024), leading to abnormal intracranial vascular tension and disrupted neurotransmitter release. This drives perivascular fluid dynamics dysregulation. The sustained release of CGRP during migraine episodes is central to inducing downstream vascular dysfunction, which stimulates GS activity by causing dilation of intracranial blood vessels (Zhang et al., 2023). However, in Zhang’s study, results indicated right-sided lateralization of abnormalities. This specificity in findings is likely attributable to population heterogeneity (the majority of migraine patients included experienced right-sided pain). Concurrently, two other studies identified right cerebral specificity, reporting extensive cortical connectivity alterations centered on the right thalamus alongside changes in neurotransmitter levels. These findings support abnormal right cerebral blood perfusion, thereby supplementing the hypothesis of right-sided pathology (Amin et al., 2018; Bathel et al., 2018). In this study, we observed a reduction in the left-sided DTI-ALPS index in the CNSP region. However, the underlying mechanisms driving this change remain unclear. This finding may also be related to the fact that all subjects included in our study were right-handed (with the majority presenting bilateral neck and shoulder pain), suggesting that the CNSP exhibits distinct pathological alterations compared to migraine. However, when observing water molecule diffusion rates in different directions, we found that along the *x*-axis of the left projection fiber region, the diffusion rate CNSP differed from HCs. Along the *y*-axis, the diffusion rate showed a positive correlation with VAS, supporting the reduced DTI-ALPS index results on the left side. This suggests that the projection fibers constitute a significant region of change for CNSP’s DTI-ALPS, which may be attributable to white matter degeneration.

The present investigation revealed a negative correlation between pain duration and DTI-ALPS, with the impairment being more pronounced in the left hemisphere; nonetheless, the underlying mechanism of this migrainous impairment remains unidentified. The limitations of DTI-ALPS may stem from its reliance on the delineated ROI, as it exclusively evaluates the overall dispersion of water molecules in the x-direction of the lateral ventricles, encompassing the medullary veins, PVS, and adjacent white matter, thereby hindering the acquisition of more nuanced information and an accurate evaluation of global signal damage throughout the brain (Taoka et al., 2024). Additionally, because different studies included various subjects, the same ROI areas might be affected by different amounts of nearby tissue pressure or functional problems (like axonal diffuse injury from traumatic brain injury). This volumetric effect (Kamagata et al., 2022), coupled with its impact on functional connectivity (Park et al., 2023), may elucidate the discrepancies in findings.

Currently, numerous approaches of GS assessment exist, each possessing distinct advantages and limitations. Intrathecal gadolinium-based contrast agent (GBCA) remains unapproved for clinical application in any nation due to its invasive characteristics, the potential danger of gadolinium encephalopathy, and the necessity for meticulous evaluation of the administered dosage (Taoka and Naganawa, 2020). Intravenous GBCA have undergone continual optimization and development, establishing IV GBCA-based MRI as the sole approved approach for contrast-enhanced MRI in human patients. Nevertheless, owing to the impact of blood flow distribution and the blood-brain barrier, additional refinement of the technique is required, including minimizing the volume effect of the blood compartment, measuring the concentration of GBCA in the brain, and establishing reference indices (Hua et al., 2025). Alongside DTI-ALPS, other non-invasive assessment modalities encompass diffusion-weighted image analysis along the perivascular space (DWI-ALPS), chemical-exchange-saturation transfer (CEST), CPV, and PVS volumetric measurements. DWI-ALPS exhibits a more rapid detection capability than DTI-ALPS, although both share similar limitations. CEST employs hydrogen atom exchange to enhance the signal of small molecules for high-resolution imaging, predominantly utilized in animal studies rather than clinical settings (Chen et al., 2017). CPV, PVS, and the fractional volume of free water (FW) in white matter have been identified as potential indicators for evaluating lymphatic injury (Zheng et al., 2025); however, their specificity is low and they are highly influenced by individual variability. This work employed DTI-ALPS to evaluate brain GS function in patients with CNSP; nonetheless, it remains in the exploratory phase, requiring further extensive validation by methods such as those mentioned above in the future.

## 5 Limitations

Several limitations should be considered when interpreting the current results. Firstly, this cross-sectional study provides preliminary insights into alterations within the GS of CNSP. Findings confirm that CNSP patients exhibit changes in GS function; however, it remains unclear whether these alterations constitute a consequence or a causative factor of CNSP. Secondly, DTI-ALPS is influenced by the manual delineation of ROIs. Although this method holds promise for mitigating the effects of head tilt and other factors, the influence of the adjacent tissue volume effect limits its ability to reflect the overall functional status of the GS (Yan et al., 2021). Furthermore, the study’s conclusions warrant cautious interpretation. Although standard methodology was employed (30 directions, b = 1,000 s/mm^2^), the suitability of this b-value as the optimal parameter for assessing free water movement within tissue remains unevaluated. Finally, this study had a limited sample size and employed only the ALPS method, failing to fully clarify the pathological mechanisms of left-sided GS dysfunction. Future research should involve large-scale clinical and basic experiments, further employing multimodal approaches to validate these findings.

## 6 Conclusion

In conclusion, our study applying DTI-ALPS, a simple and non-invasive technique, showed that DTI-ALPS indices were diminished in both the left and right cerebral hemispheres of patients with CNSP compared to the normal population, suggesting potential impact to their GS function. This phenomenon is particularly pronounced on the left side and exhibits a significant correlation with disease progression and pain intensity, indicating that CNSP onset may further compromise GS function. This finding offers new insights for deepening our understanding of this condition. Despite the limitations of our study technique, the findings undoubtedly confirm the dynamic alterations of GS damage in individuals with CNSP, which is, to a degree, beneficial for guiding clinical practice.

## Data Availability

The raw data supporting the conclusions of this article will be made available by the authors, without undue reservation.
